# Truncated FRMD7 proteins in congenital Nystagmus: novel frameshift mutations and proteasomal pathway implications

**DOI:** 10.1186/s12920-024-01817-7

**Published:** 2024-01-26

**Authors:** Yuqing Su, Juntao Zhang, Jiahui Gao, Guoqing Ding, Heng Jiang, Yang Liu, Yulei Li, Guohua Yang

**Affiliations:** 1 Department of Medical Genetics, School of Basic Medical Science, Wuhan University, Wuhan, China; 2 The First Clinical College of Wuhan University, Wuhan, China; 3 Xiangyang Central Hospital, Affiliated Hospital of Hubei University of Arts and Science, Xiangyang, 441021 China; 4 School of Basic Medicine, Hubei University of Arts and Science, Xiangyang, 441053 China; 5 Hubei Provincial Key Laboratory of Developmentally Originated Disease, Wuhan, 430071 China

**Keywords:** Congenital nystagmus, FRMD7, Novel mutation, Function validation, X-linked nystagmus

## Abstract

Idiopathic congenital nystagmus (ICN) manifests as involuntary and periodic eye movements. To identify the genetic defect associated with X-linked ICN, Whole Exome Sequencing (WES) was conducted in two affected families. We identified two frameshift mutations in *FRMD7*, c.1492dupT/p.(Y498Lfs*15) and c.1616delG/p.(R539Kfs*2). Plasmids harboring the mutated genes and qPCR analysis revealed mRNA stability, evading degradation via the NMD pathway, and corroborated truncated protein production via Western-blot analysis. Notably, both truncated proteins were degraded through the proteasomal (ubiquitination) pathway, suggesting potential therapeutic avenues targeting this pathway for similar mutations. Moreover, we conducted a comprehensive analysis, summarizing 140 mutations within the *FRMD7* gene. Our findings highlight the FERM and FA structural domains as mutation-prone regions. Interestingly, exons 9 and 12 are the most mutated regions, but 90% (28/31) mutations in exon 9 are missense while 84% (21/25) mutations in exon 12 are frameshift. A predominant occurrence of shift code mutations was observed in exons 11 and 12, possibly associated with the localization of premature termination codons (PTCs), leading to the generation of deleterious truncated proteins. Additionally, our conjecture suggests that the loss of FRMD7 protein function might not solely drive pathology; rather, the emergence of aberrant protein function could be pivotal in nystagmus etiology. We propose a dependence of FRMD7 protein normal function primarily on its anterior domain. Future investigations are warranted to validate this hypothesis.

**Supplementary Information:**

The online version contains supplementary material available at 10.1186/s12920-024-01817-7.

## Introduction

Congenital nystagmus (CN) is a group of inherited ocular disorders with an incidence of approximately 24/10,000 in the general population and 14/10,000 in infants [1]. According to the time of onset, it can be divided into congenital (within the first 6 months of life) and acquired [2]. Based on its etiology, CN can be classified into sensory-defective nystagmus (SDN) and motor-defective nystagmus or congenital motor nystagmus (CMN). SDN may result from inadequate image stimulation in the macula due to diseases of the anterior visual conduction pathway, or loss of central recess function due to organic diseases of the macula, retina, or optic nerve. It can occur in various conditions, such as congenital cataracts, aniridia, Peters’ anomaly, ocular skin albinism, color blindness, cone cell dystrophy, macular defects, congenital stationary night blindness, Leber’s congenital dark eye disease, and optic nerve hypoplasia [3, 4]. In contrast, CMN occurs in isolation without other ocular abnormalities and may be due to damage to the central nervous system or the pathways that control eye movements [5]. As a genetically heterogeneous eye movement disorder, CMN is characterized by involuntary horizontal oscillations of the eye that occur within the first 6 months of life [6]. Due to constant eye movements, it can result in reduced visual function [7], but most patients have good stereoscopic vision, and the prevalence of strabismus is 7.8-10% [8]. Other associated features may include mild visual loss, astigmatism, strabismus, and sometimes nodding movements.

CMN has been shown to be associated with multiple modes of inheritance, including autosomal dominant, autosomal recessive, and X-chromosome linked inheritance, with X-chromosome linked inheritance being the most common [9] Mutations in *FRMD7*, a member of the FERM family of proteins, associated with cytoskeletal dynamics, are the most frequent causes of X-linked ICN [10]. The *FRMD7* gene encodes a member of the FERM domain family of proteins. These are plasma membrane–cytoskeleton coupling proteins many of which bind to actin or other cytoskeleton components [11]. The *FRMD7* gene is the most predominant mutation causing idiopathic nystagmus. To date, 153 *FRMD7* mutants have been reported in the HGMD (The Human Gene Mutation Database) website.

Our identification of two mutations in the *FRMD7* gene, leading to nystagmus in two families, is of great significance. This findingreinforces the pivotal role of genetic mutations in precipitating nystagmus, underscoring the imperative for sustained inquiry in this domain. Furthermore, our comprehensive summary and analysis of mutation profiles unveil two noteworthy observations. Primarily, the FERM and FA structural domains have been found to be highly susceptible to mutations. Secondly, the prevalence of frameshift mutations predominantly within exon [12] delineates a distinct pattern. These findings offer valuable insights for the development of therapeutic advancements and deeper explorations of protein function.

## Method and materials

### Editorial policies and ethical considerations

Written informed consent was obtained from all participants of both families according to the study protocol approved by the Specialized Committee on Science Ethics of the Hubei College of Arts and Sciences under the number 2022-012 (Supplementary files 5 and 6), and the study complied with the principles of the Declaration of Helsinki.

### Patient recruitment and sample collection

Two independent family pedigrees were recruited for this study and the two affected individuals and their parents underwent genetic sequencing. Both patients underwent a thorough ophthalmologic examination by a professional ophthalmologist, which included detailed visual acuity examination, lens fissure examination, vitreous, fundus, electroretinogram (ERG), and visual evoked potential (VEP) examination. Peripheral blood samples were collected from the two affected individuals and their parents, and the *FRMD7* gene (GenBank ID: NM_194277.3) was amplified and sequenced for each subject.

### DNA sequencing and mutation analysis

Genomic DNA extracted from peripheral blood samples utilized the Blood DNA Mini kit (Simgen Biotech, Hangzhou, China), following the manufacturer’s protocol. Library construction for Whole Exome Sequencing (WES) analysis, targeting the coding regions of the human genome encompassing the FRMD7 gene, was performed (utilizing theSureSelect human all exon capture kit of Agilent).

Briefly, The process entailed purification, quantification, and library preparation of exome-enriched DNA fragments in accordance with the manufacturer’s guidelines. Sequencing was executed on the Illumina NextSeq 500 platform to acquire high-throughput sequencing data.

Data processing involved initial quality checks, adapter trimming, and removal of low-quality reads using CASAVA (1.8.2) software. The clean sequencing data were aligned to the human reference genome (GRCh37/hg19) using alignment algorithms such as BWA or Bowtie2.

Variant identification underwent cross-referencing with gnomAD to ascertain novelty and clinical significance. For mRNA splicing effect prediction, three bioinformatic tools were employed:

Human Splicing Finder (HSF): https://www.genomnis.com/.

SpliceAI: https://github.com/Illumina/SpliceAI.

Verseak: https://www.jsi-medisys.de/products/sequence-pilot/varseak/.

Prediction of protein conservation utilized:

PolyPhen2 (Polymorphism Phenotyping v2): http://genetics.bwh.harvard.edu.com.

NCBI dataset: https://www.ncbi.nlm.nih.gov/.

### Plasmid vectors construction of mutant genes

The wild-type *FRMD7* gene fragment was amplified from genomic DNA using nested PCR as the template. Two sets of primers were designed to introduce two types of mutations using overlapping extension PCR (Table 1). Plasmids from Baiyi Wuhan.

**Table 1:** PCR primer sequences

Primer	Base sequence	Length
*FRMD7-mut1-F*	ccccaggtcttttttttatgtggacaagccac	32
*FRMD7-mut1-R*	gtggcttgtccacataaaaaaaagacctgggg	32
*FRMD7-mut2-F*	gcccaaggaatatcaaatgaagagctttca	30
*FRMD7-mut2-R*	tgaaagctcttcatttgatattccttgggc	30
*pEGFP-C1-FRMD7-HindIII-F*	GCTCAAGCTTGGatgctacatttaaaagtgca	32
*pEGFP-C1-FRMD7-KpnI-R*	CCGCGGTACCttaagctaaaaagtaattac	30
*phage-FRMD7-SalI-F*	TGACGTCGACtatgctacatttaaaagtgca	31
*phage-FRMD7-NotI-R*	CGACGCGGCCGCtagctaaaaagtaattacatg	33

The PCR products were digested using restriction endonucleases KpnI and HindIII, SAII and NotI, respectively. The target gene fragment was then ligated to the vector pEGFP using T4 DNA ligase. Plasmid was introduced into the bacteria by thermal excitation. After transformation, the bacteria were cultured on antibiotic medium (AMP, KANA), and successfully transformed bacteria were screened and cultured as monoclonal. The vector primers were combined with fragment primers for PCR bacteriological examination after which the positive bacterial solution was sent for testing. The plasmid was then extracted using the Rapid Plasmid DNA Small Volume Kit (Hangzhou Xinjing Bioreagent Development Co., Ltd.).

### Cellular transient

HEK293T cells (purchased from China Center for Type Culture Collection (CCTCC)) line in good condition was used for transfection. Following resuscitation, cells were seeded into 6-well plates and cultured conventionally with complete medium overnight at 37℃ in a CO2 incubator. The plasmid and Lipofectamine transfection reagent were separately diluted with serum-free medium OPTI-MEM, with a final concentration of 4 µg/mL and 16µL/mL, respectively. The specific concentrations used for each plasmid were 500 ng/µL. Mixed and added to the wells of the plate, and incubated in the incubator for 48 h.

### qPCR

Total RNA extraction utilized the Trizol, followed by reverse transcription into cDNA using the PrimeScriptTM RT kit as per the manufacturer’s guidelines. The qPCR analysis employed SYBR Green Mix. Primers targeting the FRMD7 gene and the housekeeping gene β-action were designed (Table 2). Each condition was subjected to 3 replicated independent experiments.

data are shown as mean ± standard deviation of 3 independent experiments, *p < 0.05, **p < 0.001,****p < 0.0001(one-way ANOVA with T-test to evaluate the significance of differences observed in mRNA expression levels between the wild type and mutants.

**Table 2:** qPCR primer sequences

Primer	Base sequence	bp
*GFP-FRMD7-QPCR-F*	GTCCTGCTGGAGTTCGTGA	19
*GFP-FRMD7-QPCR-R*	agatggctgcaactcaggtt	20
*phage-FRMD7-QPCR-F*	GCCTGACGTCGACtatgcta	20
*phage-FRMD7-QPCR-R*	agatggctgcaactcaggtt	20
*β-action-F*	AGAGCTACGAGCTGCCTGAC	20
*β-action-R*	AGCACTGTGTTGGCGTACAG	20

### Western blot

HEK293T cells were transfected with pEGFP-C1-FRMD7-wt/mut1/mut2 plasmids and cultured under standard cell culture conditions. After 48 h of culture, total protein was extracted from the cells using typical RIPA lysis buffer consisted of 20mM Tris(pH 7.5), 150mM NaCl, 1% Triton X-100, and sodium pyrophosphate, β-glycerophosphate, EDTA, Na3VO4, leupeptin, sourced from Beyotime Biotechnology. The BCA Protein Quantification Kit (purchased from Shanghai Yisheng Biotechnology Co., Ltd.) was used to quantify the protein under the guidance of the instructions. The protein samples were denatured, centrifuged, and subjected to protein gel electrophoresis. The separated proteins were then transferred to nitrocellulose membranes and blocked with 5% skimmed milk for 90 min. Anti-clonal primary antibodies (from ABclonal, 1:1000) were incubated overnight, followed by incubation with secondary antibodies (from ABclonal, 1:20,000) for 1.5 h before development.

### Analysis of degradation pathways

HEK293T cells were transfected with pEGFP-C1-FRMD7-wt/mut1/mut2 plasmids and cultured under standard cell culture conditions. Upon reaching appropriate density (48 h), the cells were treated with MG-132 (a proteasome inhibitor, from MedChemExpress, 50 µM) and CQ (a lysosome inhibitor, from MedChemExpress, 50 µM) for 6 h, respectively, while a negative control NC group was also included. GAPDH was used as an internal reference. Following completion of the incubation, cells were collected, and lysis buffer was added to extract intracellular proteins. Cell debris was removed by centrifugation, and the supernatant was collected. Protein concentration was determined using the Bradford assay. The data obtained from the Western blot experiments were analyzed using gray scan ratio with imageJ and Graphpad.

## Results

### Clinical materials

We recruited two family lines with patients diagnosed with idiopathic nystagmus, and by whole exome sequencing of the candidate gene *FRMD7*, we identified two mutations in *FRMD7* in two independent families: c.1492dupT/p.(Y498Lfs*15) and c.1616delG/p.(R539Kfs*2), with the latter has never been reported in the Human Gene Mutation Database (HGMD).

**Fig. 1:**
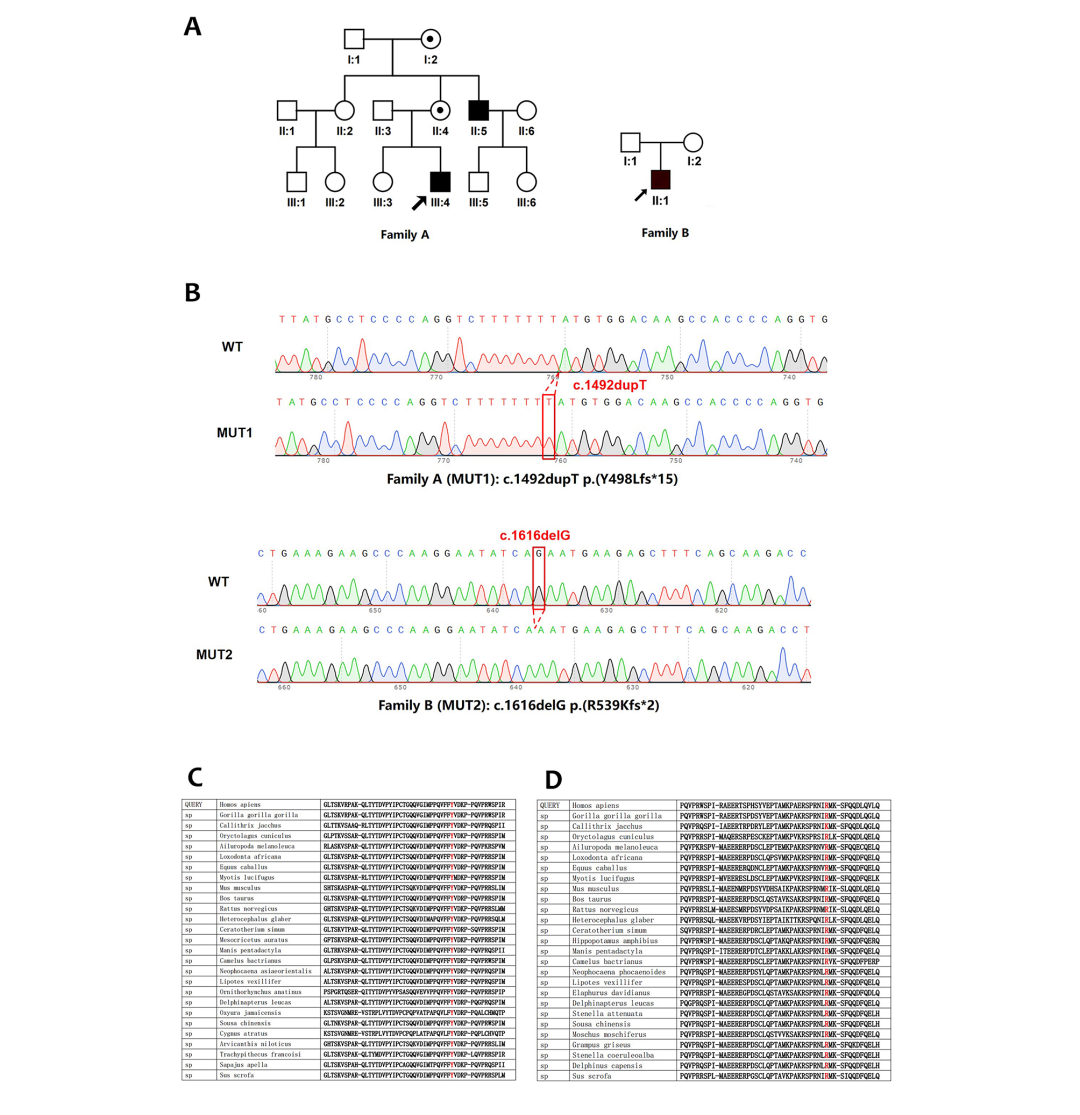
**A**: Family trees (drawn by PowerPoint2020);Family B was inherited in a typical companion X chromosome recessive manner, with male onset only. The proband (III:4) was hemizygous, his mother II:4 and grandmother I:2 were carriers, and II:5 was also hemizygous. **B**: DNA sequencing results, two mutations were found on probands: c.1492dupT/p.(Y498Lfs*15) in family A and c.1616delG/p.(R539Kfs*2) in family B. **C1**: Amino acid conservation analysis of mut1: c.1492dupT/p.(Y498Lfs*15). **C2**: Amino acid conservation analysis of mut2: c.1616delG/p.(R539Kfs*2).

The family lineage depicted in Fig. 1A illustrates the genetic inheritance pattern within the two patient families. Family A illustrates a de novo mutation scenario, observed in the proband (II:1) who exhibits involuntary eye movements, while the mother (I:2) doesn’t carry the mutated gene. Family B was inherited in a typical companion X chromosome recessive manner, with male onset only. The proband (III:4) was hemizygous, his mother II:4 and grandmother I:2 were carriers, and II:5 was also hemizygous. Both patients presented with involuntary and periodic eye movements. Mental developmental block, strabismus, photophobia, color blindness, night blindness and astigmatism were not found in any of the families and there was no occurrence of systemic abnormalities like albinism. The clinical data are summarized in Table 3. The Supplementary materials 2 and 3 include videos of patients.

**Table 3:** clinical features of probands

	Family A(III:4)	Family B(II:1)
SEX	M	M
Age(year)	4	29
Age at onset(year)	0	1
Nystagmus direction	Horizontally	Horizontally
Best corrected visual acuity at presentation(L/R)	0.3/0.3	0.3/0.2
Nystagmus features	no obvious fast or slow phase	no obvious fast or slow phase
strabismus	N	External strabismus
photophobia	N	N
color blindness	N	N
Night blindness	N	N
Astigmatism	N	N
Compensatory head position	Y	Y
Increased nystagmus when gazing	Y	Y
Reduced nystagmus when closing eyes	N	No autonomous feeling
albinism	N	N

### Identification of new mutations

Two frameshift mutations were identified in the genes of the probands, both of which are located on exon 12. Sara Hafdaoui et al. published a case report in July2023, reported the presence of a c.1492dupT mutation in the *FRMD7* gene within a nystagmus patient with Turner syndrome[12]. However, their investigation primarily encompassed genetic testing without further experimental analysis. In contrast, our study presents a more comprehensive approach as followed below, providing a deeper understanding of the mutation’s functional implications beyond genetic identification alone.

In family A, the mutation type observed in the proband (III:4) and his mother (II:4) was c.1492dupT, resulting in a substitution of tyrosine (Y) with leucine (L) at amino acid position 498. In family B, the mutation type observed in the affected individual (II:1) was c.1616delG, resulting in the substitution of arginine (R) with lysine (K) at amino acid position 539 (Fig. 1B). Both mutations lead to the early appearance of a PTC, causing the translation process to end prematurely and the production of truncated protein. By searching the NCBI database, the amino acid positions where both mutations are located are highly conserved (Fig. 1C1C2).

We also assessed the impact of two de novo mutations on mRNA splicing, namely the c.1492dupT mutation at exon 12 + 442 and the c.1616delG mutation at exon 12–530, both of which belong to the “class III mutation region” that affects splicing. Both the Varseak and SpliceAI algorithms suggest that these mutations do not affect splicing. However, the online prediction platform used for expression product prediction indicates that the mutations are “deleterious”, which implies a frameshift mutation that may lead to changes in the amino acid sequence, truncated proteins, and potential degradation of the mRNA via nonsense-mediated mRNA decay (NMD).

### qPCR detection of mRNA expression of mutant genes

**Fig. 2:**
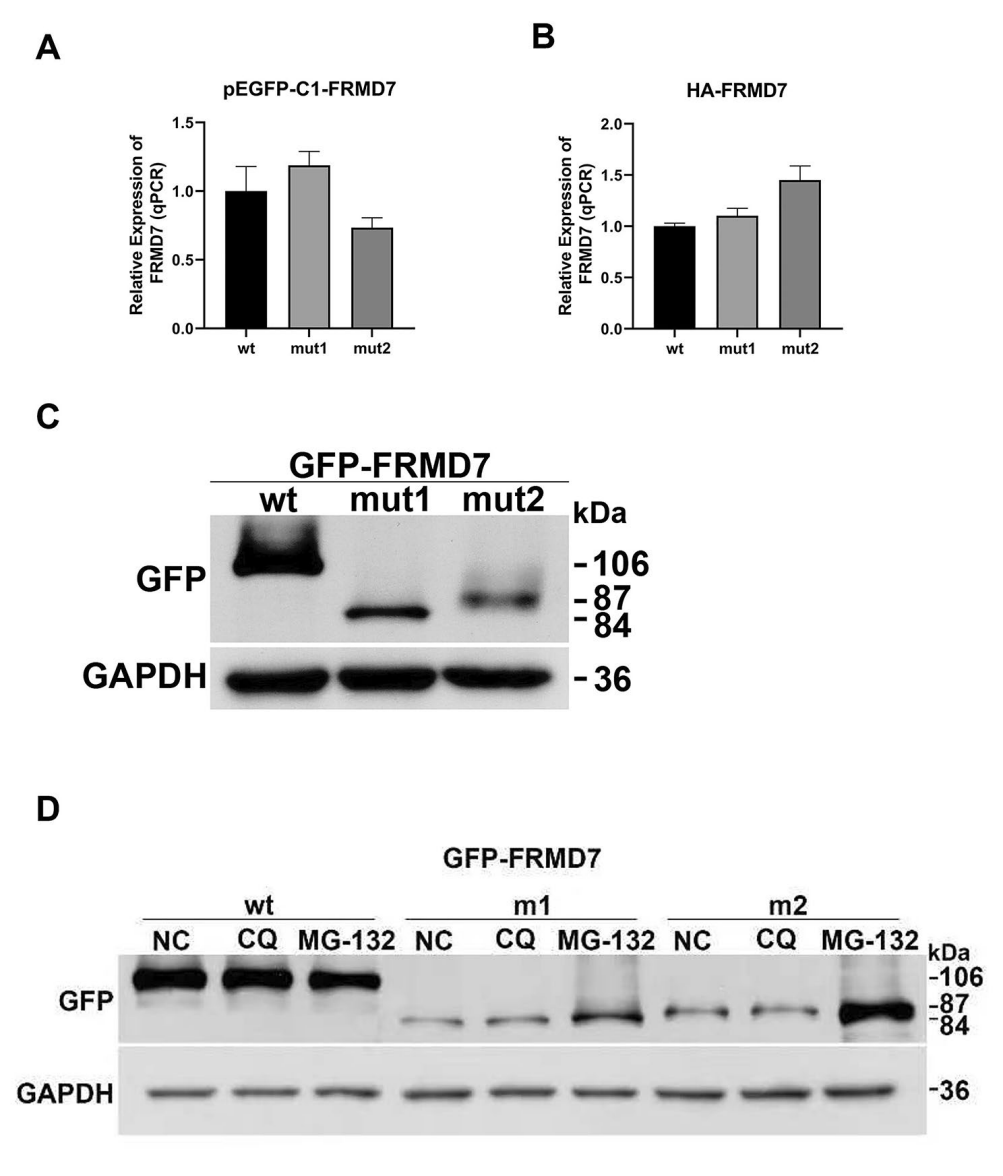
**AB**: The results of qPCR; Two groups of vectors showed no statistical difference in mRNA levels between the wild type and the mutants. **C**: Western-Blot results; The molecular weight of the mutant protein was significantly lower than that of the wild type. **D**: Results of the truncated protein degradation pathway assay. After the addition of the proteasome inhibitor MG-132, the truncated protein content increased significantly, demonstrating that both proteins were degraded via the ubiquitination pathway. (GFP: Green fluorescent protein; GAPDH: glyceraldehyde-3-phosphate dehydrogenase, serve as loading control; NC: Negative control; CQ: Chloroquine, a lysosome inhibitor; MG-132: Synonyms: Z-Leu-Leu-Leu-al, a proteasome inhibitor)

NMD is a quality control mechanism, degrading aberrant transcripts from mutated genes carrying Nonsense or shift mutations by recognizing and degrading mRNAs containing PTCs, thereby avoiding the production of truncated proteins, and thus preventing truncated proteins and potentially mitigating pathogenicity. The qPCR results (refer to Fig. 2AB) from both vectors indicated similar mRNA expression levels for c.1492dupT and c.1616delG mutations compared to the wild type, displaying no statistically significant differences. This suggests that both mutations, c.1492dupT and c.1616delG, do not significantly impact the transcription of the gene into mRNA, indicating the absence of NMD affecting the transcribed mRNA from these mutants. This phenomenon might be linked to the mutation’s location. Specifically, while the presence of PTCs generally leads to the generation of truncated proteins, not all PTCs activate the NMD pathway. When a truncation mutation occurs upstream of the splice site by > 50 nucleotides, it will be recognized and activate the nonsense-mediated decay (NMD) pathway. However, mutations occurring in the last exon or within 50 nucleotides of the splice site will not activate NMD, resulting in the generation of truncated proteins. As both of our mutations occurred in Exon 12, they are unlikely to activate the NMD pathway.

### Protein expression of mutant genes

Plasmids expressing wild-type and two mutant cDNAs were constructed and then transfected HEK293T cells. Western blot analysis was performed, and electrophoresis data supported the prediction of truncated protein production, with the wild-type FRMD7 protein having a molecular weight of 106 kDa while the mutant variants, had significantly smaller molecular weights, with mutation 1 and mutation 2 having weights of 84 and 87 kDa, respectively (Fig. 2C). (Original images in Supplementary material 7).

Abnormal FRMD7 protein expression was observed in both mutant variants, and th eir expression levels were slightly lower than the wild type which indicate that truncated protein was degraded by a certain pathway.

### Degradation of truncated proteins via the ubiquitination pathway

To delve into the degradation pathways of truncated proteins, HEK293T cells were transfected with pEGFP-C1-FRMD7-wt/mut1/mut2 plasmids and treated with MG-132 (a proteasome inhibitor) or CQ (a lysosome inhibitor). The results revealed that the normal protein expressed in the wild type did not exhibit any change in grayscale after treatment with CQ and MG-132 compared to the NC group. This indicates that the normal FRMD7 protein undergoes degradation via pathways other than the lysosomal or ubiquitination pathways.

In contrast, mutation1 (p. (Y498Lfs*15) ) displayed a significantly increase in gray scale after MG-132 treatment compared to the control group, which indicates that the degradation of truncated proteins was inhibited after the addition of proteasome inhibitors. The grayscale of the group treated with CQ was the same as that of the NC group, indicating that truncated proteins are not degraded via the lysosomal pathway. Similarly, mutation2 (p. (R539Kfs*2)) exhibited the same results as mutation1, suggesting that both truncated proteins via the ubiquitination pathway (Fig. 2D). (Original images in Supplementary material 8).

These findings carry significant implications, offering insights into in vivo pathways for abnormal protein degradation, thereby enabling us to design targeted therapeutic agents to eliminate the accumulation of abnormal proteins.

### Results of literature review of *FRMD7*

X-linked nystagmus has been linked to at least 3 different loci on the chromosome: Xp11.4-p11.3 [13]、Xq26-Xq27 [14]、Xp22.3-p22.2 [15], with *FRMD7* (Xq26-Xq27) being the most prevalent locus.

FRMD7 is a protein-coding gene comprising of 12 exons and located at Xq26.2, which encodes a protein with 714 amino acids. This gene is expressed in various tissues and organs during human embryonic development, including the midbrain, spinal cord, cerebellar primordia, ventricular layer of the forebrain, and is most abundant in the developing neural retina, vestibular system, and parts of the brain that regulate the vestibulo-ocular reflex [16, 17].Specifically, FRMD7 is expressed in a specific type of starburst neuron (Starburst Amacrine Cells) in the retina, which regulates retinal signaling and may be the molecular mechanism responsible for the oculomotor flutter following *FRMD7* mutations [18]. Calcium/calmodulin-dependent serine protein kinase (CASK) is a multi-structural domain scaffolding protein that functions in synaptic transmembrane protein anchoring and ion channel transport. It is a calcium/calmodulin-dependent serine protein kinase that does not require divalent cations for its catalytic activity. Joanne et al. demonstrated, for the first time, that knockdown of *FRMD7* during neuronal differentiation leads to altered neuronal synapse development. They found that *FRMD7* expression was upregulated in retinoic acid (RA)-induced adult neuroblastoma in differentiating NEURO2A cells in mice [19]. FRMD7 has also been shown to interact with CASK, a calcium/calmodulin-dependent serine protein kinase, which provides another clue to the pathogenesis of *FRMD7* in ICN [20]. Additionally, it has been demonstrated in animal experiments that *FRMD7* may exert nystagmogenic effects by targeting GABAAR2 [21]. As FRMD7 is homologous to FARP1 and FARP2 proteins, which are involved in synaptic growth and branching, FRMD7 protein may have similar effects [18]. More than half of the mutant phenotypes of *FRMD7* cause the appearance of nystagmus symptoms, and 1/3 are associated with idiopathic nystagmus in infants.

To date, the HGMD (The Human Gene Mutation Database) website has reported 153 different pathogenic variants of the *FRMD7* gene (Table 4), including 91 missense/nonsense variants (59.5%), 28 splicing variants (18.3%), 20 small deletions (13.0%), 7 small indels (4.6%), 1 small insertion (0.65%), and 6 gross deletions (3.9%) (HGMD professional 2023.1).

**Table 4:** Proportion of each mutation type of *FRMD7* (HGMD professional 2023.1)

Mutation type	Number	Proportion
Missense/nonsense	91	59.5%
Splicing	28	18.3%
Small deletion	20	13.0%
Small indels	7	4.6%
Small insertion	1	0.65%
Gross deletion	6	3.9%

*FRMD7* is a gene that codes for a cytosolic-cytoskeletal coupling protein, which belongs to the FERM structural domain family. The FERM structural domain family is named after its founding members, which include protein 4.1, ezrin, radixin, and moesin [22]. The protein encoded by *FRMD7* consists of a conserved FERM structural domain located at the N-terminal end and an adjacent FA structural domain (A-C or F1-F3), which forms a cloverleaf structure. The FERM structural domain is typically responsible for membrane binding through interactions with integrated membrane proteins and lipids, while the FA structural domain, present in a subset of FERM structural domain proteins, regulates protein function through modifications such as phosphorylation [11]. The FERM structural domain spans amino acids 2-282, while the FA structural domain spans amino acids 288–336. Of the 140 *FRMD7* mutants analyzed, 63 (45%) occurred in the FERM structural domain, resulting in alterations to the FERM amino acid sequence (excluding splicing abnormalities due to intron mutations), while 24 (17.1%) occurred in the FA structural domain. Mutations associated with nystagmus in *FRMD7* are highly clustered in the N-terminal region of the protein, strongly suggesting that the FERM and FA structural domains are important for the normal function of FRMD7 protein.

The FERM structural domain plays a crucial role in intracellular signaling and cytoskeleton assembly. It has the ability to interact with several other protein structural domains to regulate various physiological processes, such as cell morphology, motility, and differentiation. Mutations in the FERM structural domain have been shown to contribute to infantile nystagmus (IN) [23]. FA, the FERM-adjacent region, defines a subset of the FERM proteins in animals. The conservation of motifs in FA that are potential substrates for kinases together with the known regulatory phosphorylation of 4.1R in this region raises the possibility that FA is a regulatory adaptation in this subset of the FERM proteins [24].

**Fig. 3:**
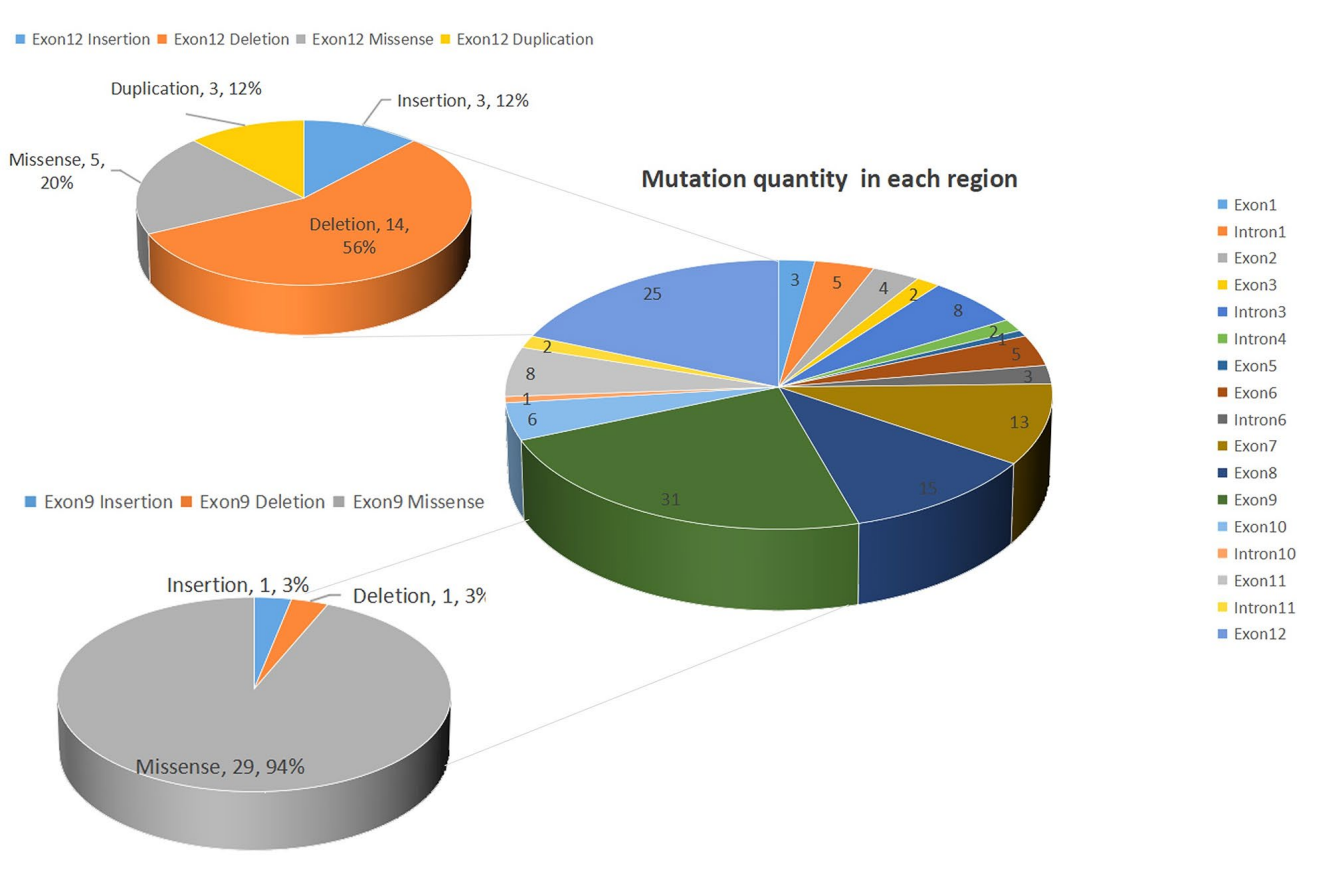
Number of mutations in each region; exon 9 was the most frequently mutated position, accounting for 31/140 (22.1%) mutations, followed by exon 12 with 25/140 (17.9%) mutations

**Fig. 4:**
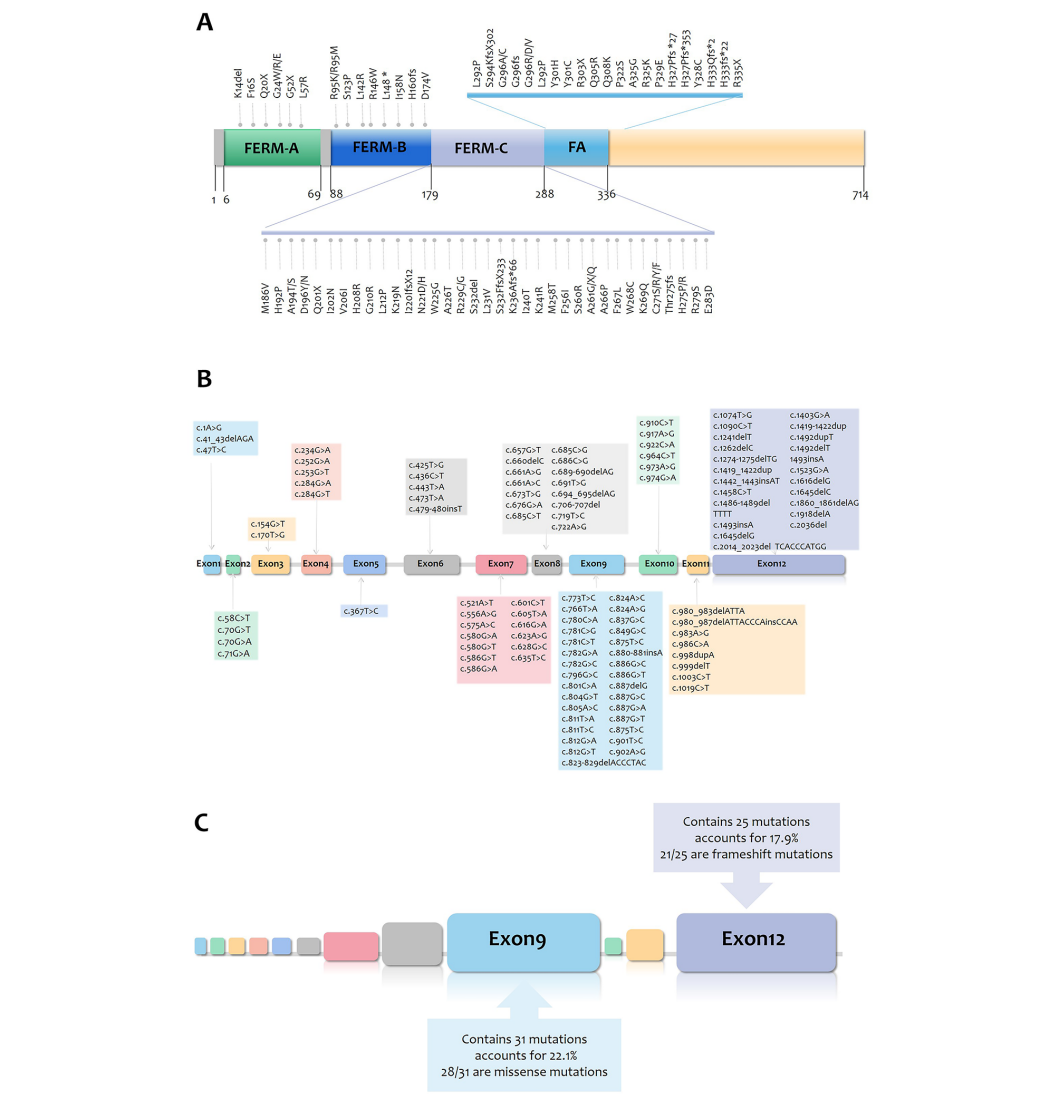
**A**: Mutation representation of FRMD7 protein in the FERM and FA structural domains. FERM is divided into three regions A/B/C and FA is the neighboring region. These four positions are hot mutation regions for mutations. **B**: Summary of mutations occurring on each exon. **C**: Exon 9 and exon 12 are the regions with the highest mutation rates, but the mutation types of the two are distinct: Exon 9 contains 31 mutations, 28 of which are missense mutations; Exon 12 contains 25 mutations, 21 of which are code-shifting mutations

We conducted a comprehensive summary of the 140 mutation types reported in the literature (Supplementary file-Table 4) [16, 17, 23, 25, 26, 27, 28, 29, 30, 31, 32, 33–36, 7, 20, 21, 37–55], including the two identified in this study. Of these mutations, 22/140 (15.7%) were found in introns, while exon 9 was the most frequently mutated position, accounting for 31/140 (22.1%) mutations, followed by exon 12 with 25/140(17.9%) mutations (Fig. 3). While missense/nonsense mutations comprise approximately 60% of total mutations, our study revealed that shift mutations accounted for up to 80% of mutations in exon 12 (Fig. 4C). This finding is noteworthy and warrants further investigation into the underlying causes.

## Discussion

Our investigation identified two novel nonsense mutations, c.1492dupT/p.(Y498Lfs15) and c.1616delG/p.(R539Kfs2), in separate families, with the latter being a newly discovered mutation. This expands the mutational spectrum of the *FRMD7* gene and provides important reference information for future genetic sequencing in patients with idiopathic nystagmus. Although neither mutation was predicted by software to affect mRNA splicing, both result in the early appearance of a PTC, leading to the production of truncated proteins with deleterious function. Interestingly, our experimental verification using qPCR and Western-blot showed that neither mutation activates the nonsense-mediated mRNA decay (NMD) pathway (Supplementary file 4). This is concerning, as truncated proteins often lack one or more functional modules or may contain abnormal functional modules, potentially exacerbating the loss of the original protein function. The incidence rate of NMD can have a significant impact on the severity of the patient’s condition. Unfortunately, neither mutation examined in this study activated the organismal NMD pathway, which may have led to more severe nystagmus and could partly explain the severity of the patient’s condition. However, other factors such as individual variability in genetic background, might also contribute to the varying severity of disease phenotype.

Further analysis highlighted that both truncated proteins are degraded via the ubiquitination pathway. These findings have significant implications for our understanding of the in vivo degradation pathways of abnormal proteins and may assist in the design of targeted therapeutic agents to eliminate the accumulation of abnormal proteins.

### Mutation hotspot area

Our comprehensive review of primary literature offered a detailed overview of mutations within the FRMD7 gene and their correlation with clinical manifestations. Our analysis revealed that the FERM protein structural domain at the N-terminal end is a hotspot for mutations, with the majority of pathogenic mutations occurring in the FERM and FA structural domains (Fig. 4A). This signifies the pivotal involvement of the FERM structural domain in the regular function of the FRMD7 protein, and highlights the need for further research to investigate the effect of these domains on FRMD7 protein function. Additionally, we found that exons 7, 8, 9, and 12 are the regions with the highest concentration of mutations (Fig. 4B), indicating that screening for mutations that cause congenital nystagmus should prioritize these exons. These findings provide valuable insights for future studies, which may lead to improved diagnosis and treatment of this inherited ocular disease.

### Frameshift mutations, NMD and truncated proteins

It is noteworthy that 80% (20/25) of the mutations identified in exon 12 were missense mutations (Fig. 4C). Interestingly, almost all of the frameshift mutations were found in exons 11 and 12, including the two frameshift mutations identified in this study. This is in stark contrast to mutations found in other exons, which are predominantly missense mutations. This phenomenon is related to the location of the code shift mutation: A truncated protein is produced when a PTC appears prematurely. There are three possible ways in which a PTC can arise [56]: 1) A missense mutation that replaces a single base pair can generate a TAG, TAA, or TGA termination codon; 2) Insertion or deletion of nucleotides that are not divisible by three can result in a frameshift, and on average, a new termination codon is generated in one out of every 20 downstream codons. 3) Mutations that cause the use of cryptic splice sites or exon-skipping often result in frameshifts, which can alter the reading frame of the genetic code and affect the protein produced. In most cases, premature termination codons are generated by frameshift mutations resulting from deletions or insertions, and premature termination codons lead to truncated protein production. Nonsense-mediated mRNA decay (NMD) is an intracellular protein quality control mechanism that recognizes PTCs and degrades aberrant mRNA, thereby preventing the production of truncated proteins. However, not all mRNAs containing PTCs can be recognized and degraded by NMD complex, but only those where the PTC is located at the beginning of the mRNA sequence, 5’ of the last intron and around 50 or more nucleotides away from the intron [57].

The reason for this is that when the PTC is too close to the intron, the mRNA degradation machinery recognizes it as a defect and degrades the entire mRNA molecule. However, if the PTC is far enough away from the intron, the mRNA degradation machinery does not recognize it as a defect and may allow some protein to be produced. Therefore, when a truncating mutation occurs in the last exon or the last 50 nucleotides of the penultimate exon, PTCs cannot be properly recognized, and NMD is not induced, leading to the production of truncated proteins. This explains why in the mutations of the *FRMD7* gene, most pathogenic mutations occur in the last 50 nucleotides of exons 12 and 11, which is a strong support for the mechanism of NMD induction.

### Truncated protein and severity of nystagmus

As we mentioned above, frameshift mutations focused on exons 11 and 12 do not induce NMD, leading to the production of truncated proteins. These truncated proteins generate additional harmful proteins, in addition to the loss of original protein function. We believe that frameshift mutations occurring at other locations will most likely be degraded through the NMD pathway, resulting in the loss of corresponding protein function. The loss of FRMD7 protein may lead to mild nystagmus due to haploinsufficiency, only the production of additional harmful truncated proteins and the generation of additional abnormal protein functions will result in severe nystagmus. This includes all frameshift mutations occurring in exon 12, all frameshift mutations in the last 50 nucleotides of exon 11, and several mutations at other locations that do not induce NMD. Considering that 80% of mutations in exon 12 are frameshift mutations that generate abnormal FRMD7 protein function, mutations at this location may lead to more severe nystagmus. The next step is to compare the relationship between different exon mutations and the degree of nystagmus in patients to validate this conclusion.

We have already explained well why frame-shift mutations are rare in regions outside exons 11 and 12, but there is still a question as to why there are few pathogenic missense mutations in these two regions, and mostly pathogenic frame-shift mutations. We speculate that this may be because a single amino acid change at the end of the FRMD7 protein has little effect on protein function, which contrasts with the significant impact of the FERM domain on protein function. In other words, FRMD7 protein function is likely mainly exerted in the front half part, and the impact of the back half is small. Further research is needed to confirm this speculation.

In conclusion, our study has contributed to enriching the mutational spectrum of the *FRMD7* gene and has further established the association between idiopathic nystagmus and the *FRMD7* gene. We have elucidated the effects of both mutations on the protein function and identified potential targeted elimination pathways of truncated proteins. However, to gain a comprehensive understanding of the molecular mechanisms underlying this inherited ocular disease, further investigation into the effects of the FERM structural domain and FA structural domain on FRMD7 protein function is warranted. These findings highlight the importance of continued research in this field, which may ultimately lead to improved diagnosis and treatment of patients with idiopathic nystagmus.

### Electronic supplementary material

Below is the link to the electronic supplementary material.

Supplementary Material 1

Supplementary Material 2

Supplementary Material 3

Supplementary Material 4

Supplementary Material 5

Supplementary Material 6

Supplementary Material 7

Supplementary Material 8

## Data Availability

DNA sequencing data is uploaded in CHINESE ACADEMY OF SCIENCES/ CHINA NATIONAL CENTER FOR BIONFORMATION with the following project number: PRJCA020873 (http://www.big.ac.cn/). The summary statistics of *FRMD7* mutation for idiopathic nystagmus can be found in NCBI. Raw data for qPCR and raw images of gel electrophoresis can be found in the Supplementary Material.

## Abbreviations

CN: Congenital nystagmus
SDN: Sensory defect nystagmus
ICN: Idiopathic congenital nystagmus
CMN: Congenital motor nystagmus
IIN: Idiopathic infantile nystagmus
PTCs: Premature termination codons
NMD: Nonsense-mediated mRNA decay
HGMD: The Human Gene Mutation Database
AMP: Ampicillin
KANA: Kanamycin
