# Supplementary material for: Truncated FRMD7 proteins in congenital Nystagmus: novel frameshift mutations and proteasomal pathway implications
Source: BMC Med Genomics. 2024 Jan 26;17:36. doi: 10.1186/s12920-024-01817-7 (PMC10811807; doi:10.1186/s12920-024-01817-7)
Supplement: Supplementary file 1 — Supplementary Material 1 [file 12920_2024_1817_MOESM1_ESM.docx]

Table 5: Summary of FRMD7 mutations

| Region | Type | Mutation | Protein change | Country | Publication |
| --- | --- | --- | --- | --- | --- |
| Exon1 | Missense | 1A>G | M1V | Korea | Choi et al.[1] |
|  | Frameshift | 41_43delAGA | K14del | England/China | Tarpey et al.[2]；Jia et al.[3] |
|  | Missense | 47T>C | F16S | England | Thomas et al.[4]; Wu et al.[5] |
| Intron 1 | Splicing | 57+1G>A | —— | China | Huang et al.[6] |
|  | Splicing | 57+2T>C | —— | China | Huang et al.[6] |
|  | Splicing | 57+5G>A | —— | Germany | Schorderet et al.[7] |
|  | Splicing | 58-1G>A | —— | England | Thomas et al.[4] |
|  | Splicing | 58-3T>A | —— | China | Huangetal.[6] |
| Exon2 | Missense | 58C>T | Q20X | Germany | Schorderetetal.[7] |
|  | Missense | 70G>T | G24W | China | Lietal.[8] |
|  | Missense | 70G>A | G24R | China/Ireland | Tarpey et al.[2] |
|  | Missense | 71G>A | G24E | Australia | Tarpey et al.[2] |
| Exon3 | Missense | 154G>T | G52X |  | Thomas et al.[9] |
|  | Missense | 170T>G | L57R | China | Huang et al.[6] |
| Intron3 | Splicing | 162+2T>C | —— | China | Huang et al.[6] |
|  | Splicing | 162+5G>A | —— | England | Tarpey et al.[2] |
|  | Splicing | 162+6T>C | —— | Korea | Choi et al.[10] |
|  | Splicing | 163-1G>T | —— | China | Hu[11] |
|  | Splicing | 205+2T>G | —— | England | Tarpey et al.[2] |
|  | Splicing | 206-1G>A | —— | China | Huang et al.[6] |
|  | Splicing | 206-2G>A | —— | China | Huang et al.[6] |
|  | Splicing | 206-5T>A | —— | China | Yan et al.[12] |
| Exon4 | Missense | 234G>A | M78I | Korea | Choi et al.[13] |
|  | Synonymous | 252G>A | V84V | England | Tarpey et al.[2] |
|  | Missense | 253G>T | D85Y | China | Huang et al.[6] |
|  | Missense | 284G>A | R95K | China | Wang et al.[14] |
|  | Missense | 284G>T | R95M | China | Bai et al.[15] |
| Intron 4 | Splicing | 284+1G>A | —— | England | Self et al.[16] |
|  | Splicing | 284+10T>G | —— | China | Wang et al.[14] |
| Exon5 | Missense | 367T>C | S123P | China | Huang et al.[6] |
| Exon6 | Missense | 425T>G | L142R | Ireland/USA | Tarpey et al.[2] |
|  | Missense | 436C>T | R146W | China | Zhang et al.[17] |
|  | Nonsense | 443T>A | L148 * | Pakistan | Arshad et al.[18] |
|  | Missense | 473T>A | I158N | China | Jia et al.[3] |
|  | Frameshift | 479-480insT | H160fs | England | Tarpey et al.[2] |
| Intron6 | Splicing | 497+5G>A | —— | Belgium | AlMoallem et al.[19] |
|  | Splicing | 498-2A>T | —— | China | Wang et al.[14] |
|  | Splicing | 498-3C>T | —— | China | Yan et al.[12] |
| Exon7 | Missense | 521A>T | D174V | China | Wang et al.[14] |
|  | Missense | 556A>G | M186V | India | Gupta et al.[20] |
|  | Missense | 575A>C | H192P | Korea | Choi et al.[10] |
|  | Missense | 580G>A | A194T | China | Jiang et al.[21] |
|  | Missense | 580G>T | A194S | China | Jia et al.[3] |
|  | Missense | 586G>T | D196Y | China | Wang et al.[14] |
|  | Missense | 586G>A | D196N | China | Bai et al.[15] |
|  | Missense | 601C>T | Q201X | Italy/Germany | Tarpey et al.[2] |
|  | Missense | 605T>A | I202N | China | Jia et al.[3] |
|  | Missense | 616G>A | V206I | China | Wang et al.[14] |
|  | Missense | 623A>G | H208R | China | Li et al.[22] |
|  | Missense | 628G>C | G210R | China | Wang et al.[14] |
|  | Missense | 635T>C | L212P | China | Liu et al.[23] |
| Intron7 | Splicing | 645+1G>C | —— | England | Tarpey et al.[2] |
| Exon8 | Missense | 657G>T | K219N | Russia | —— |
|  | Nonsense | 660delC | I220IfsX12 | England | Thomas et al.[24] |
|  | Missense | 661A>G | N221D | England | Tarpey et al.[2] |
|  | Missense | 661A>C | D221H | Korea | Choi et al.[10] |
|  | Missense | 673T>G | W225G | Switzerland | Schorderet et al.[7] |
|  | Missense | 676G>A | A226T | ENGLAND | Tarpey et al.[2] |
|  | Missense | 685C>T | R229C | China | Zhang et al.[17] |
|  | Missense | 685C>G | R229G | Turkey | Kaplan et al.[25] |
|  | Missense | 686C>G | R229G | Turkey | Kaplan et al.[2] |
|  | Frameshift | 689-690delAG | S232del | China | Li et al.[8] |
|  | Missense | 691T>G | L231V | Ireland/Germany | Tarpey et al.[2] |
|  | Frameshift | 694_695delAG | S232FfsX233 | China | Wang et al.[14] |
|  | Frameshift | 706-707del | K236Afs*66 | England | Huang et al.[6] |
|  | Missense | 719T>C | I240T | China | Zhu et al.[9] |
|  | Missense | 722A>G | K241R | Korea | Choi et al.[10] |
| Exon9 | Missense | 773T>C | M258T | China | Wang et al.[14] |
|  | Missense | 766T>A | F256I | China | Wang et al.[14] |
|  | Missense | 780C>A | S260R | China | Zhang et al.[26] |
|  | Missense | 781C>G | A261G | China | Zhang et al.[26] |
|  | Missense | 781C>T | R261X | China | Wang et al.[14] |
|  | Missense | 782G>A | R261Q | China | Li et al.[8] |
|  | Missense | 782G>C | R261G | China | Song et al.[27] |
|  | Missense | 796G>C | A266P | England | Tarpey et al.[2] |
|  | Missense | 801C>A | F267L | Belgium | AlMoallem et al.[19] |
|  | Missense | 804G>T | W268C | Russia | —— |
|  | Missense | 805A>C | K269Q | China | Wang et al.[28] |
|  | Missense | 811T>A | C271S | England | Thomas et al.[4, 5] |
|  | Missense | 811T>C | C271R | China | Wang et al.[14] |
|  | Missense | 812G>A | C271Y | Scotland | Tarpey et al.[2] |
|  | Missense | 812G>T | C271F | China | Li et al.[8]；He et al.[29] |
|  | Frameshift | 823-829delACCCTAC | Thr275fs | China | Chen et al.[9] |
|  | Missense | 824A>C | H275P | USA | Schorderet et al.[7] |
|  | Missense | 824A>G | H275R | England | Thomas et al.[24] |
|  | Missense | 837G>C | R279S | China | Wang et al.[14] |
|  | Missense | 849G>C | E283D | China | Wang et al.[14] |
|  | Missense | 875T>C | L292P | Korea | Choi et al.[10] |
|  | Frameshift | 880-881insA | S294KfsX302 | England | Self et al.[16] |
|  | Missense | 886G>C | G296A | China | Zhang et al.[26] |
|  | Missense | 886G>T | G296C | China | Xiu et al.[30] |
|  | Frameshift | 887delG | G296fs | Australia | Tarpey et al.[2] |
|  | Missense | 887G>C | G296R | China | Wang et al.[14] |
|  | Missense | 887G>A | G296D | China | Wang et al.[14] |
|  | Missense | 887G>T | G296V | China | Wang et al.[14] |
|  | Missense | 875T>C | L292P | Belgium | AlMoallem et al.[19] |
|  | Missense | 901T>C | Y301H | Korea | Rim et al.[31] |
|  | Missense | 902A>G | Y301C | England | Tarpey et al.[2] |
| Exon10 | Missense | 910C>T | R303X | China | Li et al.[8] |
|  | Missense | 917A>G | Q305R | India | Radhakrishna et al.[32] |
|  | Missense | 922C>A | Q308K | China | Wang et al.[14] |
|  | Missense | 964C>T | P322S | China | Wang et al.[14] |
|  | Missense | 973A>G | A325G | China | Jiang et al.[21] |
|  | Missense | 974G>A | R325K | China | Wang et al.[14] |
| Intron10 | Splicing | 974+2T>C | —— | China | Wang et al.[14] |
| Exon11 | Frameshift | 980_983delATTA | P329E | China | Song et al.[27] |
|  | Frameshift | 980_987delATTACCCAinsCCAA | H327Pfs *27 | China | Wang et al.[14] |
|  | Missense | 983A>G | Y328C | England | Thomas et al.[24] |
|  | Nonsense | 986C>A | H327Pfs*353 | China | Song et al.[27] |
|  | Frameshift | 998dupA | H333Qfs*2 | China | Jiang et al.[21] |
|  | Frameshift | 999delT | H333fs*22 | China | Wang et al.[14] |
|  | Missense | 1003C>T | R335X | England /India | Tarpey et al.[2] |
|  | Missense | 1019C>T | S340L | Romania | Tarpey et al.[2] |
| Intron11 | Splicing | 1050+1G>C | —— | England | Tarpey et al.[2] |
|  | Splicing | 1050+5G>A | —— | Saudi Arabia | Khan et al.[33] |
| Exon12 | Missense | 1074T>G | Y358X | China | Wang et al.[14] |
|  | Nonsense | 1090C>T | L292P | China | Zhao et al.[34] |
|  | Frameshift | 1248delT; 1299del C; and 1312delT | —— | Saudi Arabia | Galvez et al.[35] |
|  | Frameshift | 1340-2145+ 214del | —— | Saudi Arabia | Galvez et al.[35] |
|  | Frameshift | 1262delC | G296A | England | Tarpey et al.[2] |
|  | Frameshift | 1274-1275delTG | G296C | China | He et al.[29] |
|  | Frameshift | 1419_1422dup | G296fs | China | Wang et al.[36] |
|  | Frameshift | 1442_1443insAT | G296R | China | Wang et al.[14] |
|  | Nonsense | 1458C>T | G296D | China | Zhang et al.[26] |
|  | Frameshift | 1486-1489delTTTT | G296V | China | Du et al.[37] |
|  | Frameshift | 1493insA | L292P | China | Jia et al.[3] |
|  | Frameshift | 1645delG | Y301H | China | Zhang et al.[26] |
|  | Frameshift | 2014_2023del TCACCCATGG | Y301C | China | Wang et al.[36] |
|  | Missense | 1403G>A | R303X | China | Wang et al.[36] |
|  | Frameshift | 1419-1422dup | Q305R | China | Wang et al.[36] |
|  | Frameshift | 1492dupT | Q308K | China | This study |
|  | Frameshift | 1492delT | Y498Mfs*26 | Russia | —— |
|  | Frameshift | 1493insA | Y498X | China | Jia et al.[3] |
|  | Missense | 1523G>A | P322S | China | Wang et al.[36] |
|  | Frameshift | 1616delG | A325G | China | This study |
|  | Frameshift | 1645delC | V549YfsX554 | China | Zhang et al.[26] |
|  | Frameshift | 1860_1861delAG | R325K | China | Wang et al.[36] |
|  | Frameshift | 1918delA | —— | China | Wang et al.[36] |
|  | Frameshift | 2014_2023delTCACCCATGG | S672Pfs∗12 | China | Wang et al.[14] |
|  | Frameshift | 2036del | L679Rfs*8 | Belgium | AlMoallem et al.[19] |

1 Sarvananthan, N. *et al.* The prevalence of nystagmus: the Leicestershire nystagmus survey. *Investigative ophthalmology & visual science* **50**, 5201-5206, doi:10.1167/iovs.09-3486 (2009).

2 Papageorgiou, E., McLean, R. J. & Gottlob, I. Nystagmus in childhood. Pediatrics and neonatology 55, 341-351, doi:10.1016/j.pedneo.2014.02.007 (2014).

3 Forssman, B. & Ringnér, B. Prevalence and inheritance of congenital nystagmus in a Swedish population. *Annals of human genetics* **35**, 139-147 (1971).

4 Stayte, M., Reeves, B. & Wortham, C. Ocular and vision defects in preschool children. *The British journal of ophthalmology* **77**, 228-232, doi:10.1136/bjo.77.4.228 (1993).

5 Jacobs, J. B. & Dell'Osso, L. F. Congenital nystagmus: hypotheses for its genesis and complex waveforms within a behavioral ocular motor system model. *Journal of vision* **4**, 604-625, doi:10.1167/4.7.7 (2004).

6 Casteels, I., Harris, C. M., Shawkat, F. & Taylor, D. Nystagmus in infancy. *The British journal of ophthalmology* **76**, 434-437, doi:10.1136/bjo.76.7.434 (1992).

7 AlMoallem, B. *et al.* Novel FRMD7 Mutations and Genomic Rearrangement Expand the Molecular Pathogenesis of X-Linked Idiopathic Infantile Nystagmus. *Investigative ophthalmology & visual science* **56**, 1701-1710, doi:10.1167/iovs.14-15938 (2015).

8 Thomas, S. *et al.* Phenotypical characteristics of idiopathic infantile nystagmus with and without mutations in FRMD7. *Brain : a journal of neurology* **131**, 1259-1267, doi:10.1093/brain/awn046 (2008).

9 Oetting, W. S., Armstrong, C. M., Holleschau, A. M., DeWan, A. T. & Summers, G. C. Evidence for genetic heterogeneity in families with congenital motor nystagmus (CN). *Ophthalmic genetics* **21**, 227-233 (2000).

10 Pu, J. *et al.* Nystagmus-related FRMD7 gene influences the maturation and complexities of neuronal processes in human neurons. *Brain and behavior* **9**, e01473, doi:10.1002/brb3.1473 (2019).

11 Diakowski, W., Grzybek, M. & Sikorski, A. F. Protein 4.1, a component of the erythrocyte membrane skeleton and its related homologue proteins forming the protein 4.1/FERM superfamily. *Folia histochemica et cytobiologica* **44**, 231-248 (2006).

12 Cabot, A. *et al.* A gene for X-linked idiopathic congenital nystagmus (NYS1) maps to chromosome Xp11.4-p11.3. *American journal of human genetics* **64**, 1141-1146, doi:10.1086/302324 (1999).

13 Kerrison, J. B., Vagefi, M. R., Barmada, M. M. & Maumenee, I. H. Congenital motor nystagmus linked to Xq26-q27. *American journal of human genetics* **64**, 600-607, doi:10.1086/302244 (1999).

14 Liu, J. Y. *et al.* Identification of a novel GPR143 mutation in a large Chinese family with congenital nystagmus as the most prominent and consistent manifestation. *Journal of human genetics* **52**, 565-570, doi:10.1007/s10038-007-0152-3 (2007).

15 Tarpey, P. *et al.* Mutations in FRMD7, a newly identified member of the FERM family, cause X-linked idiopathic congenital nystagmus. *Nature genetics* **38**, 1242-1244, doi:10.1038/ng1893 (2006).

16 Thomas, M. G. *et al.* The clinical and molecular genetic features of idiopathic infantile periodic alternating nystagmus. *Brain : a journal of neurology* **134**, 892-902, doi:10.1093/brain/awq373 (2011).

17 Yonehara, K. *et al.* Congenital Nystagmus Gene FRMD7 Is Necessary for Establishing a Neuronal Circuit Asymmetry for Direction Selectivity. *Neuron* **89**, 177-193, doi:10.1016/j.neuron.2015.11.032 (2016).

18 Betts-Henderson, J. *et al.* The nystagmus-associated FRMD7 gene regulates neuronal outgrowth and development. *Human molecular genetics* **19**, 342-351, doi:10.1093/hmg/ddp500 (2010).

19 Wang, Z., Wang, M., Wang, C. & Lu, B. Identification and functional characterization of a novel missense mutation in FRMD7 responsible for idiopathic congenital nystagmus. *Acta biochimica et biophysica Sinica* **51**, 178-184, doi:10.1093/abbs/gmy161 (2019).

20 Jiang, L. *et al.* FRMD7 Mutations Disrupt the Interaction with GABRA2 and May Result in Infantile Nystagmus Syndrome. *Investigative ophthalmology & visual science* **61**, 41, doi:10.1167/iovs.61.5.41 (2020).

21 Chishti, A. H. *et al.* The FERM domain: a unique module involved in the linkage of cytoplasmic proteins to the membrane. *Trends in biochemical sciences* **23**, 281-282, doi:10.1016/s0968-0004(98)01237-7 (1998).

22 Huang, L. *et al.* Correlations of FRMD7 gene mutations with ocular oscillations. *Sci Rep* **12**, 9914, doi:10.1038/s41598-022-14144-7 (2022).

23 Baines, A. J. A FERM-adjacent (FA) region defines a subset of the 4.1 superfamily and is a potential regulator of FERM domain function. *BMC genomics* **7**, 85, doi:10.1186/1471-2164-7-85 (2006).

24 Choi, J. H., Shin, J. H., Seo, J. H., Jung, J. H. & Choi, K. D. A start codon mutation of the FRMD7 gene in two Korean families with idiopathic infantile nystagmus. *Sci Rep* **5**, 13003, doi:10.1038/srep13003 (2015).

25 Jia, X. *et al.* Novel mutations of FRMD7 in Chinese patients with congenital motor nystagmus. *Molecular medicine reports* **16**, 1753-1758, doi:10.3892/mmr.2017.6824 (2017).

26 Wu, S. *et al.* A Disease-Causing FRMD7 Variant in a Chinese Family with Infantile Nystagmus. *Journal of molecular neuroscience : MN* **67**, 418-423, doi:10.1007/s12031-018-1245-5 (2019).

27 Schorderet, D. F. *et al.* Novel mutations in FRMD7 in X-linked congenital nystagmus. Mutation in brief #963. Online. *Human mutation* **28**, 525, doi:10.1002/humu.9492 (2007).

28 Li, N. *et al.* Five novel mutations of the FRMD7 gene in Chinese families with X-linked infantile nystagmus. *Molecular vision* **14**, 733-738 (2008).

29 Zhu, Y. *et al.* Identifcation of a novel mutation p.I240T in the FRMD7 gene in a family with congenital nystagmus. *Sci Rep* **3**, 3084, doi:10.1038/srep03084 (2013).

30 Choi, J. H. *et al.* Genotype and Phenotype Spectrum of FRMD7-Associated Infantile Nystagmus Syndrome. *Investigative ophthalmology & visual science* **59**, 3181-3188, doi:10.1167/iovs.18-24207 (2018).

31 Hu, Y. *et al.* A novel splicing mutation of the FRMD7 gene in a Chinese family with X-linked congenital nystagmus. *Molecular vision* **18**, 87-91 (2012).

32 Yan, N. *et al.* X-linked inheritances recessive of congenital nystagmus and autosomal dominant inheritances of congenital cataracts coexist in a Chinese family: a case report and literature review. *BMC medical genetics* **20**, 41, doi:10.1186/s12881-019-0780-4 (2019).

33 Choi, J. H. *et al.* Diagnostic yield of targeted next-generation sequencing in infantile nystagmus syndrome. *Ophthalmic genetics* **42**, 561-569, doi:10.1080/13816810.2021.1938138 (2021).

34 Wang, X. F. *et al.* Genotype-Phenotype Analysis and Mutation Spectrum in a Cohort of Chinese Patients With Congenital Nystagmus. *Frontiers in cell and developmental biology* **9**, 627295, doi:10.3389/fcell.2021.627295 (2021).

35 Bai, D. *et al.* Clinical feature and waveform in infantile nystagmus syndrome in children with FRMD7 gene mutations. *Science China. Life sciences* **60**, 707-713, doi:10.1007/s11427-017-9089-5 (2017).

36 Self, J. E. *et al.* Allelic variation of the FRMD7 gene in congenital idiopathic nystagmus. *Archives of ophthalmology (Chicago, Ill. : 1960)* **125**, 1255-1263, doi:10.1001/archopht.125.9.1255 (2007).

37 Zhang, Q., Xiao, X., Li, S. & Guo, X. FRMD7 mutations in Chinese families with X-linked congenital motor nystagmus. *Molecular vision* **13**, 1375-1378 (2007).

38 Arshad, M. W. *et al.* FRMD7 Gene Alterations in a Pakistani Family Associated with Congenital Idiopathic Nystagmus. *Genes* **14**, doi:10.3390/genes14020346 (2023).

39 Gupta, S. *et al.* A novel mutation in FRMD7 causes X-linked idiopathic congenital nystagmus in a North Indian family. *Neuroscience letters* **597**, 170-175, doi:10.1016/j.neulet.2015.04.037 (2015).

40 Li, N. et al. Investigation of the gene mutations in two Chinese families with X-linked infantile nystagmus. Molecular *vision* **17**, 461-468 (2011).

41 Liu, Z. *et al.* A novel missense mutation in the FERM domain containing 7 (FRMD7) gene causing X-linked idiopathic congenital nystagmus in a Chinese family. *Molecular vision* **19**, 1834-1840 (2013).

42 Thomas, M. G. *et al.* Abnormal retinal development associated with FRMD7 mutations. *Human molecular genetics* **23**, 4086-4093, doi:10.1093/hmg/ddu122 (2014).

43 Kaplan, Y. *et al.* Skewed X inactivation in an X linked nystagmus family resulted from a novel, p.R229G, missense mutation in the FRMD7 gene. *The British journal of ophthalmology* **92**, 135-141, doi:10.1136/bjo.2007.128157 (2008).

44 Zhang, X. *et al.* Identification of three novel mutations in the FRMD7 gene for X-linked idiopathic congenital nystagmus. *Sci Rep* **4**, 3745, doi:10.1038/srep03745 (2014).

45 Song, F. W. *et al.* Novel mutation c.980_983delATTA compound with c.986C>A mutation of the FRMD7 gene in a Chinese family with X-linked idiopathic congenital nystagmus. *Journal of Zhejiang University. Science. B* **14**, 479-486, doi:10.1631/jzus.B1200259 (2013).

46 He, X. *et al.* A novel frameshift mutation in FRMD7 causing X-linked idiopathic congenital nystagmus. *Genetic testing* **12**, 607-613, doi:10.1089/gte.2008.0070 (2008).

47 Xiu, Y. *et al.* Identification of a novel idiopathic congenital nystagmus‑causing missense mutation, p.G296C, in the FRMD7 gene. *Molecular medicine reports* **18**, 2816-2822, doi:10.3892/mmr.2018.9260 (2018).

48 Rim, J. H. *et al.* Accuracy of Next-Generation Sequencing for Molecular Diagnosis in Patients With Infantile Nystagmus Syndrome. *JAMA ophthalmology* **135**, 1376-1385, doi:10.1001/jamaophthalmol.2017.4859 (2017).

49 Radhakrishna, U. *et al.* Novel homozygous, heterozygous and hemizygous FRMD7 gene mutations segregated in the same consanguineous family with congenital X-linked nystagmus. *European journal of human genetics : EJHG* **20**, 1032-1036, doi:10.1038/ejhg.2012.60 (2012).

50 Khan, A. O., Shinwari, J., Al-Sharif, L., Khalil, D. S. & Al Tassan, N. Prolonged pursuit by optokinetic drum testing in asymptomatic female carriers of novel FRMD7 splice mutation c.1050 +5 G>A. *Archives of ophthalmology (Chicago, Ill. : 1960)* **129**, 936-940, doi:10.1001/archophthalmol.2011.166 (2011).

51 Zhao, H. *et al.* Molecular genetic analysis of patients with sporadic and X-linked infantile nystagmus. *BMJ open* **6**, e010649, doi:10.1136/bmjopen-2015-010649 (2016).

52 Galvez-Ruiz, A., Galindo-Ferreiro, A. & Lehner, A. J. A new gene mutation in a family with idiopathic infantile nystagmus. *Saudi journal of ophthalmology : official journal of the Saudi Ophthalmological Society* **35**, 61-65, doi:10.4103/1319-4534.325787 (2021).

53 Wang, F., Guan, H., Liu, W., Zhao, G. & Liu, S. Next-generation sequencing identifies a novel frameshift variant in FRMD7 in a Chinese family with idiopathic infantile nystagmus. *Journal of clinical laboratory analysis* **34**, e23012, doi:10.1002/jcla.23012 (2020).

54 Du, W. *et al.* A novel frame-shift mutation in FRMD7 causes X-linked idiopathic congenital nystagmus in a Chinese family. *Molecular vision* **17**, 2765-2768 (2011).

55 Byers, P. H. Killing the messenger: new insights into nonsense-mediated mRNA decay. *The Journal of clinical investigation* **109**, 3-6, doi:10.1172/jci14841 (2002).

56 Lewis, B. P., Green, R. E. & Brenner, S. E. Evidence for the widespread coupling of alternative splicing and nonsense-mediated mRNA decay in humans. *Proc Natl Acad Sci U S A* **100**, 189-192, doi:10.1073/pnas.0136770100 (2003).

1. Choi JH, Shin JH, Seo JH, Jung JH, Choi KD: **A start codon mutation of the FRMD7 gene in two Korean families with idiopathic infantile nystagmus**. *Sci Rep* 2015, **5**:13003.

2. Tarpey P, Thomas S, Sarvananthan N, Mallya U, Lisgo S, Talbot CJ, Roberts EO, Awan M, Surendran M, McLean RJ *et al*: **Mutations in FRMD7, a newly identified member of the FERM family, cause X-linked idiopathic congenital nystagmus**. *Nature genetics* 2006, **38**(11):1242-1244.

3. Jia X, Zhu X, Li Q, Jia X, Li S, Guo X: **Novel mutations of FRMD7 in Chinese patients with congenital motor nystagmus**. *Molecular medicine reports* 2017, **16**(2):1753-1758.

4. Thomas MG, Crosier M, Lindsay S, Kumar A, Thomas S, Araki M, Talbot CJ, McLean RJ, Surendran M, Taylor K *et al*: **The clinical and molecular genetic features of idiopathic infantile periodic alternating nystagmus**. *Brain : a journal of neurology* 2011, **134**(Pt 3):892-902.

5. Wu S, Deng S, Song Z, Xu H, Yang Z, Liu X, Qi L, Deng H, Yuan L: **A Disease-Causing FRMD7 Variant in a Chinese Family with Infantile Nystagmus**. *Journal of molecular neuroscience : MN* 2019, **67**(3):418-423.

6. Huang L, Zhou Y, Chen W, Lin P, Xie Y, He K, Zhang S, Wu Y, Li N: **Correlations of FRMD7 gene mutations with ocular oscillations**. *Sci Rep* 2022, **12**(1):9914.

7. Schorderet DF, Tiab L, Gaillard MC, Lorenz B, Klainguti G, Kerrison JB, Traboulsi EI, Munier FL: **Novel mutations in FRMD7 in X-linked congenital nystagmus. Mutation in brief #963. Online**. *Human mutation* 2007, **28**(5):525.

8. Li N, Wang L, Cui L, Zhang L, Dai S, Li H, Chen X, Zhu L, Hejtmancik JF, Zhao K: **Five novel mutations of the FRMD7 gene in Chinese families with X-linked infantile nystagmus**. *Molecular vision* 2008, **14**:733-738.

9. Zhu Y, Zhuang J, Ge X, Zhang X, Wang Z, Sun J, Yang J, Gu F: **Identifcation of a novel mutation p.I240T in the FRMD7 gene in a family with congenital nystagmus**. *Sci Rep* 2013, **3**:3084.

10. Choi JH, Jung JH, Oh EH, Shin JH, Kim HS, Seo JH, Choi SY, Kim MJ, Choi HY, Lee C *et al*: **Genotype and Phenotype Spectrum of FRMD7-Associated Infantile Nystagmus Syndrome**. *Investigative ophthalmology & visual science* 2018, **59**(7):3181-3188.

11. Hu Y, Shen J, Zhang S, Yang T, Huang S, Yuan H: **A novel splicing mutation of the FRMD7 gene in a Chinese family with X-linked congenital nystagmus**. *Molecular vision* 2012, **18**:87-91.

12. Yan N, Xiao L, Hou C, Guo B, Fan W, Deng Y, Ma K: **X-linked inheritances recessive of congenital nystagmus and autosomal dominant inheritances of congenital cataracts coexist in a Chinese family: a case report and literature review**. *BMC medical genetics* 2019, **20**(1):41.

13. Choi JH, Kim SJ, Thomas MG, Jung JH, Oh EH, Shin JH, Cho JW, Kim HS, Park JY, Choi SY *et al*: **Diagnostic yield of targeted next-generation sequencing in infantile nystagmus syndrome**. *Ophthalmic genetics* 2021, **42**(5):561-569.

14. Wang XF, Chen H, Huang PJ, Feng ZK, Hua ZQ, Feng X, Han F, Xu XT, Shen RJ, Li Y *et al*: **Genotype-Phenotype Analysis and Mutation Spectrum in a Cohort of Chinese Patients With Congenital Nystagmus**. *Frontiers in cell and developmental biology* 2021, **9**:627295.

15. Bai D, Shi W, Qi Z, Li W, Wei A, Cui Y, Li C, Li L: **Clinical feature and waveform in infantile nystagmus syndrome in children with FRMD7 gene mutations**. *Science China Life sciences* 2017, **60**(7):707-713.

16. Self JE, Shawkat F, Malpas CT, Thomas NS, Harris CM, Hodgkins PR, Chen X, Trump D, Lotery AJ: **Allelic variation of the FRMD7 gene in congenital idiopathic nystagmus**. *Archives of ophthalmology (Chicago, Ill : 1960)* 2007, **125**(9):1255-1263.

17. Zhang Q, Xiao X, Li S, Guo X: **FRMD7 mutations in Chinese families with X-linked congenital motor nystagmus**. *Molecular vision* 2007, **13**:1375-1378.

18. Arshad MW, Shabbir MI, Asif S, Shahzad M, Leydier L, Rai SK: **FRMD7 Gene Alterations in a Pakistani Family Associated with Congenital Idiopathic Nystagmus**. *Genes* 2023, **14**(2).

19. AlMoallem B, Bauwens M, Walraedt S, Delbeke P, De Zaeytijd J, Kestelyn P, Meire F, Janssens S, van Cauwenbergh C, Verdin H *et al*: **Novel FRMD7 Mutations and Genomic Rearrangement Expand the Molecular Pathogenesis of X-Linked Idiopathic Infantile Nystagmus**. *Investigative ophthalmology & visual science* 2015, **56**(3):1701-1710.

20. Gupta S, Pathak E, Chaudhry VN, Chaudhry P, Mishra R, Chandra A, Mukherjee A, Mutsuddi M: **A novel mutation in FRMD7 causes X-linked idiopathic congenital nystagmus in a North Indian family**. *Neuroscience letters* 2015, **597**:170-175.

21. Jiang L, Li Y, Yang K, Wang Y, Wang J, Cui X, Mao J, Gao Y, Yi P, Wang L *et al*: **FRMD7 Mutations Disrupt the Interaction with GABRA2 and May Result in Infantile Nystagmus Syndrome**. *Investigative ophthalmology & visual science* 2020, **61**(5):41.

22. Li N, Wang X, Wang Y, Wang L, Ying M, Han R, Liu Y, Zhao K: **Investigation of the gene mutations in two Chinese families with X-linked infantile nystagmus**. *Molecular vision* 2011, **17**:461-468.

23. Liu Z, Mao S, Pu J, Ding Y, Zhang B, Ding M: **A novel missense mutation in the FERM domain containing 7 (FRMD7) gene causing X-linked idiopathic congenital nystagmus in a Chinese family**. *Molecular vision* 2013, **19**:1834-1840.

24. Thomas MG, Crosier M, Lindsay S, Kumar A, Araki M, Leroy BP, McLean RJ, Sheth V, Maconachie G, Thomas S *et al*: **Abnormal retinal development associated with FRMD7 mutations**. *Human molecular genetics* 2014, **23**(15):4086-4093.

25. Kaplan Y, Vargel I, Kansu T, Akin B, Rohmann E, Kamaci S, Uz E, Ozcelik T, Wollnik B, Akarsu NA: **Skewed X inactivation in an X linked nystagmus family resulted from a novel, p.R229G, missense mutation in the FRMD7 gene**. *The British journal of ophthalmology* 2008, **92**(1):135-141.

26. Zhang X, Ge X, Yu Y, Zhang Y, Wu Y, Luan Y, Sun J, Qu J, Jin ZB, Gu F: **Identification of three novel mutations in the FRMD7 gene for X-linked idiopathic congenital nystagmus**. *Sci Rep* 2014, **4**:3745.

27. Song FW, Chen BB, Sun ZH, Wu LP, Zhao SJ, Miao Q, Tang XJ: **Novel mutation c.980_983delATTA compound with c.986C>A mutation of the FRMD7 gene in a Chinese family with X-linked idiopathic congenital nystagmus**. *Journal of Zhejiang University Science B* 2013, **14**(6):479-486.

28. Wang Z, Wang M, Wang C, Lu B: **Identification and functional characterization of a novel missense mutation in FRMD7 responsible for idiopathic congenital nystagmus**. *Acta biochimica et biophysica Sinica* 2019, **51**(2):178-184.

29. He X, Gu F, Wang Z, Wang C, Tong Y, Wang Y, Yang J, Liu W, Zhang M, Ma X: **A novel frameshift mutation in FRMD7 causing X-linked idiopathic congenital nystagmus**. *Genetic testing* 2008, **12**(4):607-613.

30. Xiu Y, Yao Y, Yang T, Pan M, Yang H, Fang W, Gu F, Zhao J, Zhu Y: **Identification of a novel idiopathic congenital nystagmus‑causing missense mutation, p.G296C, in the FRMD7 gene**. *Molecular medicine reports* 2018, **18**(3):2816-2822.

31. Rim JH, Lee ST, Gee HY, Lee BJ, Choi JR, Park HW, Han SH, Han J: **Accuracy of Next-Generation Sequencing for Molecular Diagnosis in Patients With Infantile Nystagmus Syndrome**. *JAMA ophthalmology* 2017, **135**(12):1376-1385.

32. Radhakrishna U, Ratnamala U, Deutsch S, Bartoloni L, Kuracha MR, Singh R, Banwait J, Bastola DK, Johar K, Nath SK *et al*: **Novel homozygous, heterozygous and hemizygous FRMD7 gene mutations segregated in the same consanguineous family with congenital X-linked nystagmus**. *European journal of human genetics : EJHG* 2012, **20**(10):1032-1036.

33. Khan AO, Shinwari J, Al-Sharif L, Khalil DS, Al Tassan N: **Prolonged pursuit by optokinetic drum testing in asymptomatic female carriers of novel FRMD7 splice mutation c.1050 +5 G>A**. *Archives of ophthalmology (Chicago, Ill : 1960)* 2011, **129**(7):936-940.

34. Zhao H, Huang XF, Zheng ZL, Deng WL, Lei XL, Xing DJ, Ye L, Xu SZ, Chen J, Zhang F *et al*: **Molecular genetic analysis of patients with sporadic and X-linked infantile nystagmus**. *BMJ open* 2016, **6**(4):e010649.

35. Galvez-Ruiz A, Galindo-Ferreiro A, Lehner AJ: **A new gene mutation in a family with idiopathic infantile nystagmus**. *Saudi journal of ophthalmology : official journal of the Saudi Ophthalmological Society* 2021, **35**(1):61-65.

36. Wang F, Guan H, Liu W, Zhao G, Liu S: **Next-generation sequencing identifies a novel frameshift variant in FRMD7 in a Chinese family with idiopathic infantile nystagmus**. *Journal of clinical laboratory analysis* 2020, **34**(1):e23012.

37. Du W, Bu J, Dong J, Jia Y, Li J, Liang C, Si S, Wang L: **A novel frame-shift mutation in FRMD7 causes X-linked idiopathic congenital nystagmus in a Chinese family**. *Molecular vision* 2011, **17**:2765-2768.
