# Supplementary figures and images for: Truncated FRMD7 proteins in congenital Nystagmus: novel frameshift mutations and proteasomal pathway implications
Source: BMC Med Genomics. 2024 Jan 26;17:36. doi: 10.1186/s12920-024-01817-7 (PMC10811807; doi:10.1186/s12920-024-01817-7)

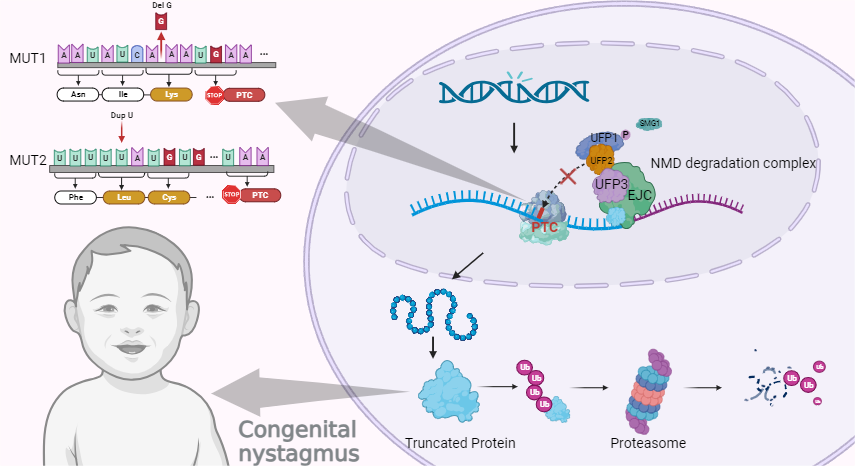

Supplement: Supplementary file 4 — Supplementary Material 4 [file 12920_2024_1817_MOESM5_ESM.png]

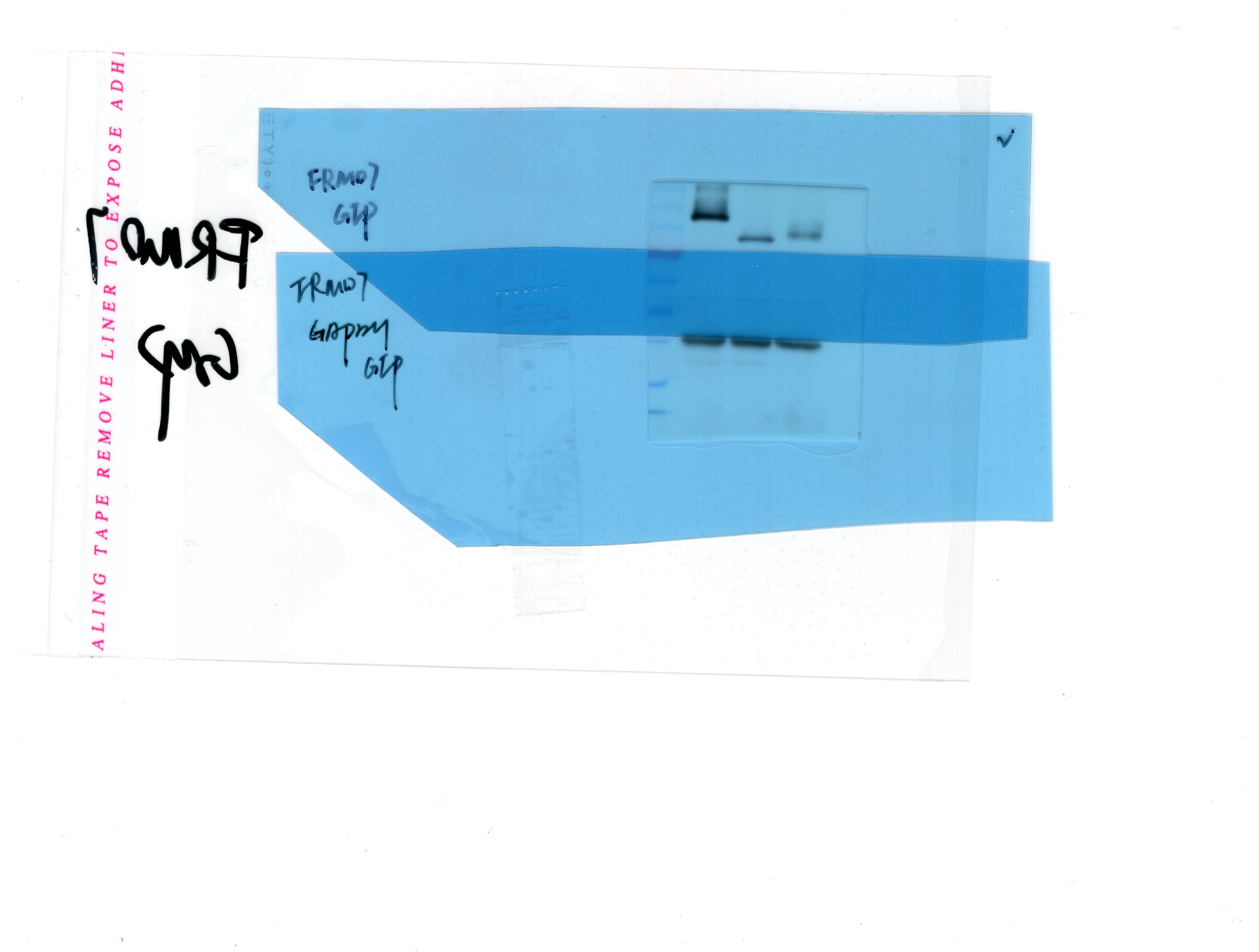

Supplement: Supplementary file 7 — Supplementary Material 7 [file 12920_2024_1817_MOESM9_ESM.jpg]

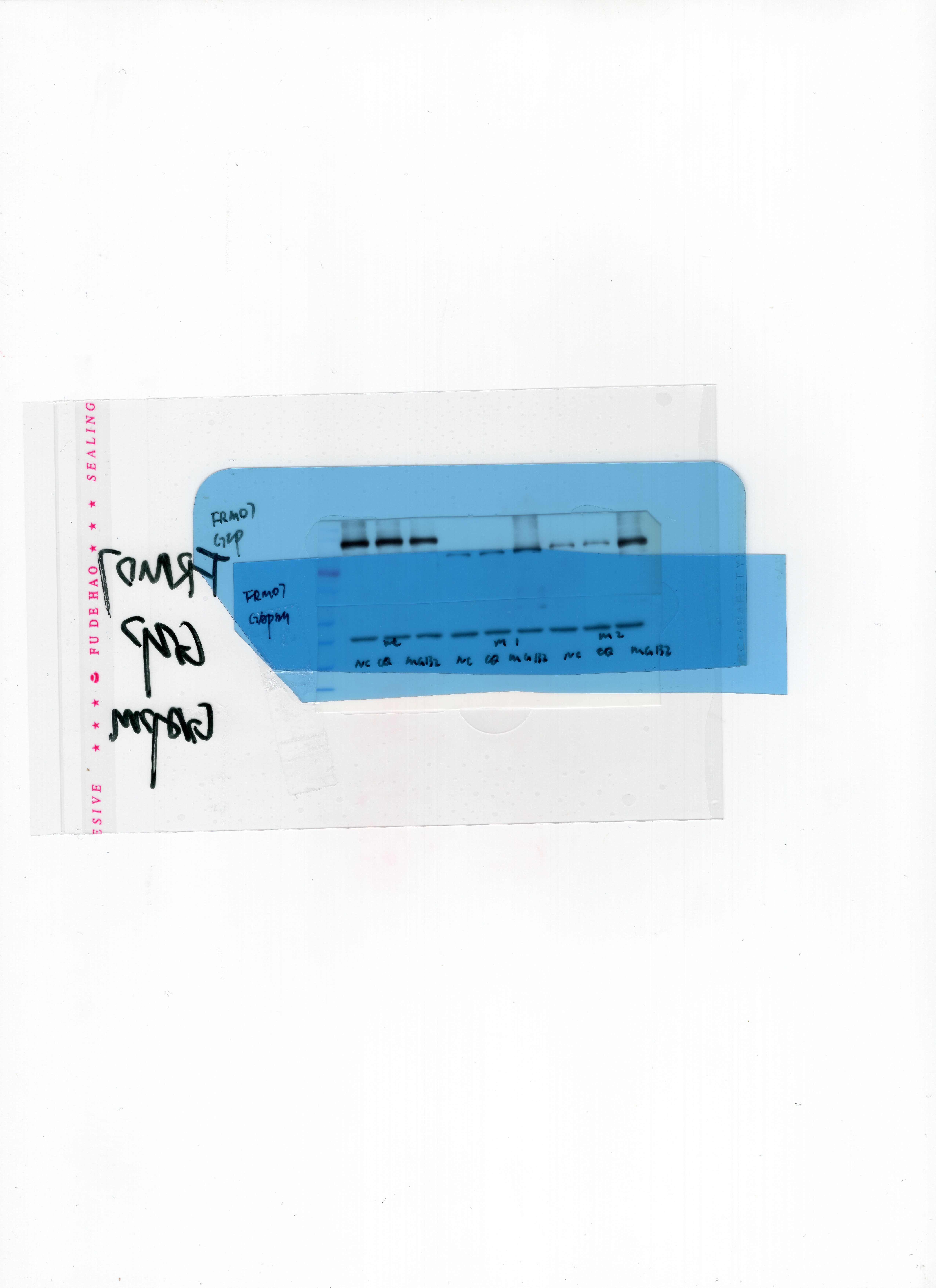

Supplement: Supplementary file 8 — Supplementary Material 8 [file 12920_2024_1817_MOESM10_ESM.jpg]
