# Supplementary material for: Truncated FRMD7 proteins in congenital Nystagmus: novel frameshift mutations and proteasomal pathway implications
Source: BMC Med Genomics. 2024 Jan 26;17:36. doi: 10.1186/s12920-024-01817-7 (PMC10811807; doi:10.1186/s12920-024-01817-7)
Supplement: Supplementary file 6 — Supplementary Material 6 [file 12920_2024_1817_MOESM8_ESM.pdf]

罕见病研究知情同意书

湖北文理学院医学部遗传与罕见病研究协作组，长期从事人类神经系统和眼科等重大遗传病的分子病理机理研究。近期，因科研需要，欲采集200例先天性眼球震颤患者及相关亲属外周血样本5毫升进行突变分析和相应的功能研究。该项目对理解先天性眼球震颤的致病机制、开发新的积极有效的基因诊断方法及个体化治疗有很大帮助。受试者在了解此情况后，参加试验是自愿的，可以拒绝参加或在试验的任何阶段随时退出而不会遭到歧视或报复，其医疗待遇与权益不受任何影响。试验期间，受试者可以随时了解与其相关的信息资料。所有研究人员将尊重患者的隐私权，为患者保守一切秘密，若公开发表实验结果，也会对受试者的身份保密。

受试者签字 徐若云　　　　2022 年 11 月 17 日

研究者签字 李雨雷　　　　2022 年 11 月 23 日
